# Evaluating the Impact of Thermal Processing on the Anti-Inflammatory Activity of Non-Centrifugal Cane Sugar: Implications on Cytokine Secretion and TLR4 Signaling

**DOI:** 10.3389/fphar.2022.905347

**Published:** 2022-06-28

**Authors:** Laura Rueda-Gensini, Julian A. Serna, Natalia I. Bolaños, Jader Rodriguez, Juan C. Cruz, Carolina Muñoz-Camargo

**Affiliations:** ^1^ Department of Biomedical Engineering, School of Engineering, Universidad de los Andes, Bogotá, Colombia; ^2^ Vice-presidency of Research and Creation, Universidad de los Andes, Bogotá, Colombia; ^3^ Centro de Investigación Tibaitatá, Corporación Colombiana de Investigación Agropecuaria, Mosquera, Colombia

**Keywords:** non-centrifugal sugar, anti-inflammatory, immunomodulation, thermal processing, TLR4 signaling

## Abstract

Plant-derived products have gained considerable attention as inflammation modulators given the wide variety of anti-inflammatory phytochemicals reported to be present in plants and their limited side effects *in vivo* during prolonged exposure periods. Non-centrifugal cane sugar (NCS) has been identified as a promising sugarcane-derived product due to its high polyphenolic composition and antioxidant potential, but its incorporations into nutraceuticals and other relevant products of biomedical interest has been limited by the ample composition-wise variability resulting from extreme and loosely controlled processing conditions. Here, we assessed the effect of reducing thermal exposure during NCS processing on the retained polyphenolic profiles, as well as on their antioxidant and anti-inflammatory activities. Specifically, we proposed two modified NCS production methods that reduce exposure to unwanted thermal processing conditions by *1*) limiting the employed temperatures through vacuum-aided dehydration and *2*) by reducing exposure time through refractance window evaporation. By comparing the modified NCS products with traditional NCS, we showed that the proposed process strategies yield enhanced polyphenolic profiles, as evidenced by the results of the Folin-Ciocalteu polyphenol quantification method and the components identification by HPLC coupled to mass spectrometry. Although these compositional differences failed to impact the antioxidant profiles and cytocompatibility of the products, they showed an enhanced anti-inflammatory potential, given their superior modulation capacity of inflammatory cytokine secretion in both systemic and neuroinflammatory scenarios *in vitro*. Moreover, we showed that both modified NCS products interfere with TLR4 signaling in human monocytes to a significantly greater extent than traditional NCS. However, the anti-inflammatory effect of NCS produced under window refractance evaporation was slightly superior than under vacuum-aided dehydration, demonstrating that reducing exposure time to high temperatures is likely more effective than reducing the operation temperature. Overall, these findings demonstrated that limiting thermal exposure is beneficial for the development of NCS-based natural products with superior anti-inflammatory potential, which can be further exploited in the rational design of more potent nutraceuticals for potentially preventing chronic inflammatory diseases.

## Introduction

Inflammation is an evolutionarily conserved process directed towards protecting the host against invading pathogens by controlling the propagation of infection and promoting tissue recovery (Netea et al., 2017; Furman et al., 2019). By eliciting the recruitment and activation of the immune system, inflammatory responses are central for triggering defense and repair mechanisms. However, certain environmental, psychological, social, and biological factors have been described to prevent the resolution of acute inflammation, thereby leading to prolonged low-grade, non-infective chronic inflammation (Calder et al., 2013). This excessive and persistent inflammatory response can, in turn, exacerbate tissue damage by propagating highly oxidative environments and by inducing major alterations both at the cellular and organ levels (Kotas and Medzhitov, 2015; Sim et al., 2020). Ultimately, this inflammatory imbalance promotes the breakdown of immune tolerance overtime and increases the risk of developing chronic disorders such as hypertension (Furman et al., 2017), diabetes (Jin et al., 2013) and cancer (Taniguchi and Karin, 2018), as well as cardiovascular (Gisterå & Hansson, 2017), autoimmune (Straub and Schradin, 2016) and neurodegenerative diseases (Heneka et al., 2014).

Accordingly, there is an increasing interest in the discovery of anti-inflammatory agents with the therapeutic potential of neutralizing excessive and persistent inflammatory reactions (Jeong et al., 2014). In this regard, a variety of steroidal and non-steroidal drugs with potent anti-inflammatory activities have been developed, but their reported side effects during prolonged exposure periods have considerably limited their clinical administration (del Grossi Moura et al., 2018). A special focus has been therefore given to the development of plant-derived dietary supplements, which exhibit much fewer side effects and whose long-term administration could modulate immune responses by exploiting the wide variety of immunomodulating phytochemicals found in plants (Jantan et al., 2015). Sugarcane (*Saccharum officinarum*), in particular, has attracted considerable attention by researchers worldwide due to the abundance of polyphenolic compounds (e.g., phenolic acids, flavonoids and glycosides) in sugarcane juices and unrefined products derived from its processing (Singh et al., 2015). This rich polyphenolic profile has been correlated to exceptional antioxidant activities, as well as a broad range of therapeutic activities such as analgesic, antithrombotic and anti-inflammatory (Jaffé, 2012; Singh et al., 2015). In fact, several of these medicinal benefits have been traditionally observed in widely consumed sugarcane’s unrefined products such as non-centrifugal cane sugar (NCS), which can preserve close phytochemical profiles to native sugarcane given their minimal processing (Singh et al., 2015; Velásquez et al., 2019). However, this natural product’s therapeutic potential remains practically untapped in the context of functional foods and nutraceuticals, mainly due to substantial composition-wise variability resulting from diverging environmental and unfavorable production conditions (Pandey and Rizvi, 2009; Robles-Sánchez et al., 2009; Rodrigues et al., 2009; Hussain et al., 2016; Velásquez et al., 2019). In particular, the excessive and uncontrolled exposure to extreme thermal processing conditions during traditional NCS production may be a major contributor to the thermally induced degradation of sugarcane’s volatile bioactive compounds. This leads to reduced biochemical profiles and suboptimal biological performance (Robles-Sánchez et al., 2009; Rodrigues et al., 2009; Velásquez et al., 2019).

This work is, therefore, aimed towards thoroughly characterizing the impact of reducing exposure to extreme thermal processing conditions on the anti-inflammatory activities of NCS. For this, we propose two alternative NCS production methods that reduce thermal exposure during the conversion of sugarcane juice into NCS: *1*) vacuum-aided concentration, which reduces the temperature necessary for evaporating the aqueous content, and *2*) refractance window evaporation, which facilitates rapid heat transfer and considerably reduces the exposure time to high temperatures. We compare the polyphenolic composition of the NCS products obtained by the two alternative methods with traditionally produced NCS and evaluate how this reflects on their anti-inflammatory activities. Specifically, we investigate the impact of these modified NCS products on pro-inflammatory and anti-inflammatory cytokine secretion in stimulated immune cells, as well as on the expression of important targets along toll-like receptor 4 (TLR4)-induced inflammatory pathways. Modulating TLR4 signaling is of particular interest for targeting chronic inflammatory disorders, considering that it plays a pivotal role in the propagation of inflammatory environments by orchestrating the production of pro-inflammatory mediators (Lucas & Maes, 2013). With this study, we intend to contribute to optimizing NCS production to obtain natural products with high nutraceutical value, especially in the prevention of chronic inflammatory disorders.

## Materials and Methods

### Materials

2,2-diphenyl-1-picryl-hydrazyl-hydrate (DPPH) was purchased from ChemCruz Biochemicals (Santa Cruz Biotechnology, Dallas, TX, United States). 2,2′-azino-bis (3-ethylbenzothiazoline-6-sulphonic acid) (ABTS), potassium persulfate, formic acid, ammonium formate, acetonitrile and 3-(4,5-dimethylthiazol-2-yl)- 2,5- diphenyltetrazolium bromide (MTT), dimethyl sulfoxide (DMSO) and the Follin ciocalteu reagent were purchased from Sigma-Aldrich (San Luis, MO, United States). Dulbecco’s modified Eagle’s medium (DMEM), RPMI 1640 medium and fetal bovine serum (FBS) were purchased from Biowest (Kansas City, MO, United States). Trypsin-EDTA was purchased from Gibco (Thermo Scientific, Waltham, MA, United States). Triton X-100 was purchased from Cell Biolabs Inc. (San Diego, CA, United States). Reference standards for analytical HPLC-MS: caffeine (Part No. C8960-250G, Sigma–Aldrich), theobromine (Part No. T4500-25G, Sigma–Aldrich), theophylline (Part No. T1633-25G, Sigma–Aldrich), (±)-catechin (C) (Part No. C1788-500MG, Sigma–Aldrich), (−)-epigallocatechin gallate (EGCG) (Part No. E4143-50MG, Sigma–Aldrich), (−)-epicatechin (EC) (Part No. E1753-1G, Sigma–Aldrich), (−)-epicatechin gallate (ECG) (Part No. E3893-10MG, Sigma–Aldrich), (−)-epigallocatechin (EGC) (Part N E3768-5MG, Sigma–Aldrich), caffeic acid (Part No. C0625, Sigma–Aldrich), p-coumaric acid (Part No. C9008, Sigma–Aldrich), rosmarinic acid (Part No. 536954-5G, Sigma–Aldrich), quercetin (Part N Q4951-10G, Sigma–Aldrich), naringenin (Part No. N5893-1G, Sigma–Aldrich), luteolin (Part No. L9283-10MG, Sigma–Aldrich), kaempferol (Part No. K0133-50MG, Sigma–Aldrich), ursolic acid (Part N° U6753-100MG; SigmaAldrich), pinocembrin (Part No. P5239, Sigma–Aldrich), carnosic acid (Part N° C0609-10MG; Sigma-Aldrich), apigenin (Part No. A3145-25MG, Sigma–Aldrich), 3-rutinoside cyanidin (Part No. G36428; Sigma–Aldrich), pelargonidin 3-glucoside (Part No. 53489; Sigma–Aldrich), quercetin 3-glucoside (Part No. 89230; Phytolab), kaempferol 3-glucoside (Part N° 89237; Phytolab).

### Sample Obtention and Processing

**FIGURE 1:**
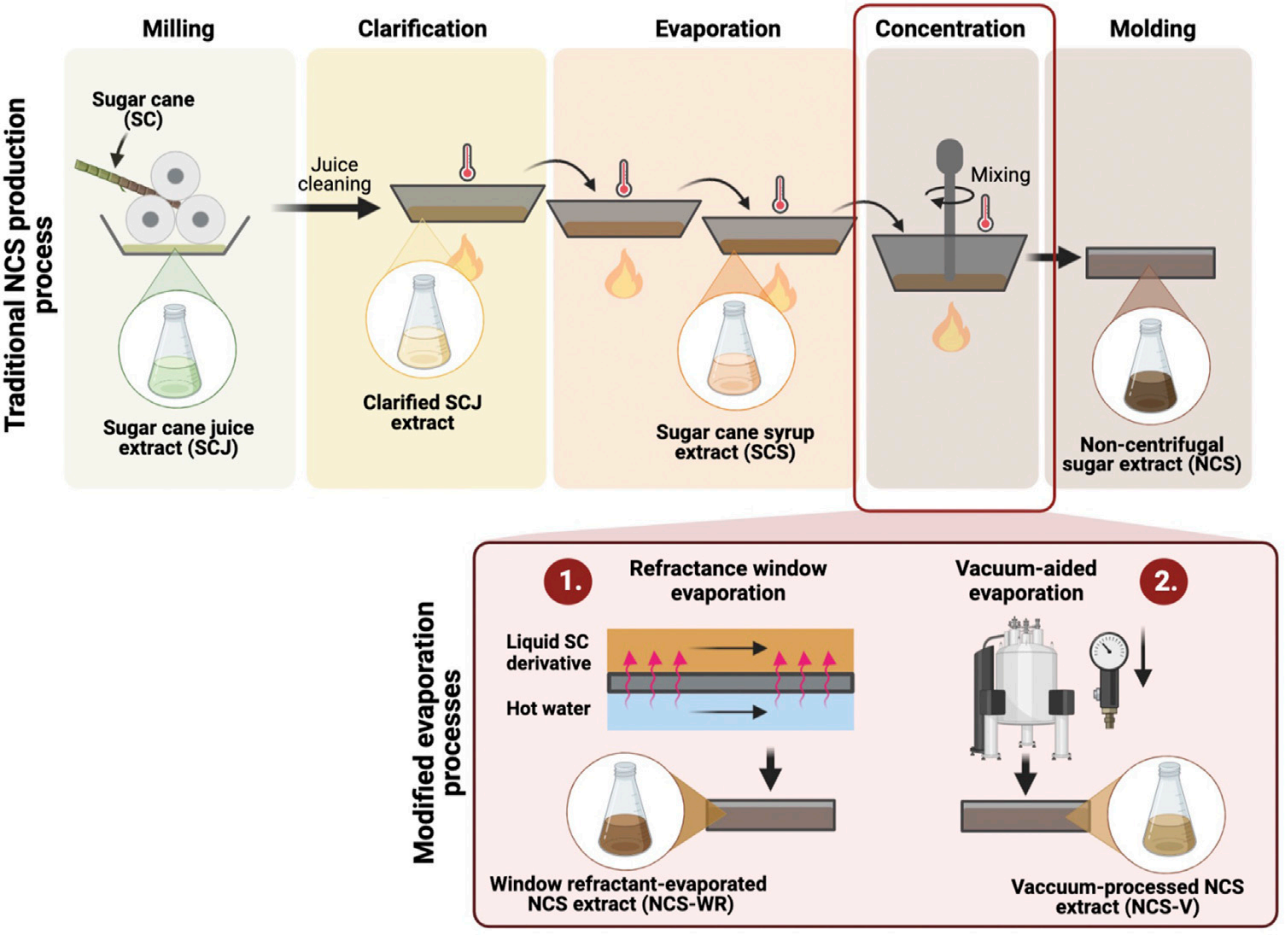
General schematic of traditional NCS production, as well as the two modified concentration processes that employ (1) refractance window evaporation and (2) vacuum-aided evaporation. Created with BioRender.com.

The Colombian Corporation of Agropecuarian Research (Corporación Colombiana de Investigación Agropecuaria, Agrosavia) provided samples from four different stages of the traditional NCS production process described in Figure 1. Specifically, the samples were collected immediately after sugarcane milling (sugarcane juice), after juice clarification (clarified sugarcane juice), during clarified juice dehydration (sugarcane syrup) and of the final NCS product. These samples are henceforth termed SCJ, C-SCJ, SCS and NCS, respectively. In addition, samples of NCS obtained from the two modified production processes, namely vacuum-aided and refractance window evaporation, were also collected and termed NCS-V and NCS-WR. All samples were produced from sugarcane grown under the same edaphic and environmental conditions.

#### Vacuum-Aided Evaporation

Sugarcane syrup (SCS) at approximately 70° Brix was concentrated up to 94° Brix using a vacuum-aided evaporator system that operated under vacuum conditions (−0.735 to −0.27 atm), temperature ranges between 54 and 80°C and a vapor pressure of 50 psi. The evaporator had a hemispherical bottom and was equipped with a stirrer that enabled a much more efficient heat exchange.

#### Refractance Window Evaporation

Sugarcane syrup (SCS) at approximately 70° Brix was also concentrated up to 94° Brix using refractance window evaporation, a technique designed to dry heat-sensitive products while limiting temperature exposure time (Bernaert et al., 2019). SCS was applied uniformly on a polyethylene film that moved simultaneously over a hot water surface (90–95°C), which enabled the transfer of sensible heat through the film using infrared radiation and rapid water evaporation.

### Methanolic Extraction of Polyphenolic Compounds

The free polyphenolic compounds of all sugarcane-derived samples were isolated through methanolic solid-liquid extraction following a modified version of the protocol reported by Duarte-Almeida et al. (Duarte-Almeida et al., 2011). Briefly, all samples were lyophilized and mixed at a 1:1 solid (g)-liquid (ml) ratio with pure methanol using a T25 digital Ultra Turrax homogenizer (IKA, Staufen, Germany) at 12,000 RPM for 60 s. The soluble phase was then collected through vacuum filtration while the insoluble one was resuspended in the same volume of methanol as described above. This process was repeated twice. The methanol from the extracted soluble phases was removed with rotary evaporation (Hei-VAP, Heidolph, Schwabach, Germany) under vacuum conditions, 37°C and 80 RPM, and the dry content was subsequently resuspended in ddH_2_O at a 1:2.5 ratio with respect to the original dry weight of each sample. In this case, 50 ml of ddH_2_O were used to resuspend the extracted content of 20 g of the original sample dry weight. All extracts were stored at 4°C and protected from light until further use. The extraction process was performed in triplicate for each sample.

### Quantification of Polyphenolic Content

The total polyphenolic content of each sugarcane-derived extract was determined through the Folin-Ciocalteu method (Lamuela-Raventós, 2017). Briefly, 50 µl of each sample, previously diluted with ddH_2_O at a 1:8 ratio, was further diluted in 2.95 ml of ddH_2_O. This solution was then mixed with 250 µl of the Folin-Ciocalteu reagent and incubated for 8 min at room temperature. Seven hundred and fifty microliters of 3.5% (w/v) sodium carbonate were then added and completed to 5 ml with ddH_2_O. The mixture was incubated at 37°C for 30 min and finally, the absorbance was measured at 750 nm aided by a spectrophotometer (ThermoFisher Scientific, Waltham, MA, United States). A gallic acid standard curve was also performed up to 800 mg/L to estimate the gallic acid equivalent polyphenolic content of each sample (Supplementary Figure S1A).

### Polyphenolic Compound Identification With Analytical HPLC-Coupled to Mass Spectrometry

The principal polyphenolic compounds in each sugarcane-derived extract were identified through high-performance liquid chromatography (HPLC) coupled to mass spectrometry, following a protocol described previously by Cifuentes et al. (Cifuentes et al., 2021). The extracts were dissolved in a 0.2% methanol:water solution in formic acid (1:1), followed by vortexing for 5 and 5 min of ultrasonication. Samples were first separated with a Dionex Ultimate 3000 UHPLC system (Thermo Scientific, Sunnyvale, CA, United States) equipped with a binary gradient pump (HP G3400RS), a sample automatic injector (WPS 300TRS) and a temperature controlled Hypersil GOLD Aq column (TCC 3000; Thermo Scientific) that was kept at 30°C during the run. This column was coupled to an Orbitrap mass spectrometer (Exactive Plus, Thermo Scientific) equipped with an ion current detection system to identify the separated compounds. The two mobile phases employed for the gradient elution were (A) an aqueous solution of 0.2% (v/v) formic acid and (B) acetonitrile with 0.2% (w/v) ammonium formate. These were mixed following a gradient program starting at 100% phase A for 4 min, then changing it linearly to 100% phase B during 8 min, and returning to starting conditions for 1 min. Mass spectra were acquired in a mass range between m/z 60–900 under full scan mode. Identification of polyphenolic compounds was determined by comparing retention time and mass measurements (Δppm <0.001) with reference standards. Results are presented as concentration of respective polyphenol per kg of dry sample and the corresponding error propagation analysis as estimated from the instruments precision (%).

### Radical Scavenging Potential

#### DPPH Assay

DPPH is a stable free radical commonly used for screening radical scavenging activity due to its easily detectable violet appearance in its oxidized state and its colorless appearance in its reduced state. The radical scavenging activity of sugarcane-derived extracts was evaluated by assessing its interaction with DPPH, following a modified version of the protocol reported by Boly et al. (Boly et al., 2016). Briefly, an equal volume of each extract was mixed with a 0.2 mM methanolic DPPH solution in a 96-well microplate. The dilution ratio was varied between 1:8 to 1:692. After 30 min of incubation in the dark, the absorbance was measured at 490 nm with a microplate reader (ThermoFisher Scientific). A DPPH solution in the absence of extracts was used as a negative control. The DPPH radical scavenging (RS) percentage of each extract concentration was calculated by following Eq. 1:

$$\text{RS }\left(\text{\%}\right)=\frac{{\text{A}}_{\text{C}}-{\text{A}}_{\text{S}}}{{\text{A}}_{\text{C}}}*100$$ (1)

where A_C_ is the absorbance of the control and A_S_ is the absorbance of the sample. Experiments for each condition were performed in triplicate.

#### ABTS Assay

The scavenging of the ABTS radical (ABTS^+^) in the presence of sugarcane-derived extracts was also determined following a modified version of the protocol reported by Re et al. (Re et al., 1999). Briefly, ABTS was converted into its radical cation by mixing 7 mM ABTS with 2.4 mM potassium persulfate at a 1:1 volume ratio overnight. This ABTS^+^ stock solution was diluted with 60% (v/v) methanol until reaching a 0.713 ± 0.002 absorbance at 734 nm excitation. Different dilutions of each extract, ranging from 1:8 to 1:692, were then mixed with the diluted ABTS^+^ solution at a 1:7 volume ratio and allowed to react in the dark for 5 min. Absorbance was measured at 734 nm with a UV-Vis spectrophotometer (ThermoFisher Scientific). An ABTS^+^ solution in the absence of extracts was used as negative control and the radical scavenging percentage of the assayed samples was also calculated according to Eq. 1. Each sample and controls were tested in triplicate.

Ascorbic acid standard curves were also performed in the presence of each radical to determine the ascorbic acid equivalent scavenging potential of the extracts (Supplementary Figures S1B,C). In this regard, the standard curves were used to determine the concentration of ascorbic acid that yielded the same radical scavenging percentage of extracts at a fixed dilution ratio, and this was then normalized by the original sample concentration.

### Cell Culture

THP-1 human acute leukemia monocytes (TIB-202^™^, ATCC) were maintained in RPMI 1640 medium supplemented with 10% (v/v) FBS and 0.05 mM 2-mercaptoethanol at a cellular density ranging from 100,000 cells/ml to 500,000 cells/ml. Cells were not allowed to exceed this density range to avoid any unwanted activation processes prior to conducting the inflammation studies. RAW 264.7 murine macrophages (TIB-71^™^, ATCC) were maintained in DMEM supplemented with 10% (v/v) FBS, at cellular densities below 80% confluency. Cells were detached during subculturing by incubating them with 5mM EDTA at 37°C for 10 min, followed by gentle scrapping. Vero cells (CCL-81^™^, ATCC) were maintained in DMEM supplemented with 5% (v/v) FBS. All cells were cultured under a humidified atmosphere at 37°C and 5% CO_2_ concentration.

### Cytocompatibility

Cell viability in response to sugarcane-derived extracts was determined by quantifying metabolic activity *via* the colorimetric MTT assay. Oxidoreductase enzymes in viable cells reduce the tetrazolium dye MTT to insoluble formazan, which has a characteristic violet color that can be measured spectrophotometrically. Accordingly, Vero and RAW264.7 cells were seeded in 96-well plates at a density of 10,000 cells per well, while or THP-1 cells were seeded at a density of 20,000 cells per well. After 24 h, they were exposed to serial dilutions of each extract in the range of 1:8 to 1:192 for another 24 and 48 h. After the respective incubation time, the MTT reagent was added to each well at a final concentration of 0.5 mg/ml and subsequently incubated for 2 h. Cells were then lysed with pure DMSO, and the absorbance was measured at 595 nm. Untreated cells were used as positive control (C^+^), while cells previously lysed with 10% (v/v) Triton X-100 were used as negative control (C^−^). Cell viability was determined according to Eq. 2.

$$\text{MTT viability }\left(\text{\%}\right)=\frac{{\text{A}}_{\text{S }}-{\text{ A}}_{{\text{C}}^{-}}}{{\text{A}}_{{\text{C}}^{+}}-{\text{ A}}_{{\text{C}}^{-}}}\text{∗}100$$ (2)

The membrane permeability of Vero cells was also determined by quantifying the leakage of lactate dehydrogenase (LDH) upon exposure to all extracts. In this case, 50 µl of the medium of treated cells after respective incubation times was collected and mixed with 50 µl of the LDH reagent (Roche, Basilea, Switzerland) and incubated for 30 min at room temperature. Absorbance was then measured at 490 nm and viability according to LDH leakage was quantified by following Eq. 3, using the same positive and negative controls as described above.

$$\text{LDH viability }\left(\text{\%}\right)=\left(1-\frac{{\text{A}}_{\text{S }}-{\text{ A}}_{{\text{C}}^{-}}}{{\text{A}}_{{\text{C}}^{+}}-{\text{ A}}_{{\text{C}}^{-}}}\right)\text{∗}100$$ (3)

### Intracellular ROS Quantification

The effect of sugarcane-derived extracts on intracellular reactive oxygen species (ROS) production was determined in α-synuclein stimulated THP-1 monocytes before and after exposure. ROS production was quantified with the cell permeant reagent 2′,7′ –dichlorofluorescin diacetate (DCFDA), which fluoresces in the presence of hydroxyl, peroxyl and other ROS, using the commercial DCFDA/H2DCFDA-Cellular ROS Assay Kit (Abcam, Cambridge, United Kingdom). THP-1 monocytes were concentrated to 1.0 × 10^6^ cells/ml in 10% (v/v) FBS supplemented 1X Buffer and exposed to 20 µM DCFDA for 30 min at 37°C. Cells were then washed with supplemented 1X Buffer and simultaneously treated with 10 μg/ml A53T α-synuclein (S1071; Sigma-Aldrich) and each extract diluted at a 1:8 ratio in the same buffer. After 4 h, fluorescence was quantified at Ex/Em = 485/535 nm with a fluorescence plate reader (FluoroMax Spectrofluorimeter, Horiba Scientific, Kyoto, Japan). A53T α-synuclein stimulated monocytes without extracts were used as a positive control and unstimulated and untreated monocytes were used as negative controls.

### Induction of Inflammation Scenarios

#### Modeling Systemic Inflammation

Bacterial infections were modeled *in vitro* to simulate immune responses in systemic inflammation scenarios using *1*) *Escherichia coli* lipopolysaccharide (LPS) (LPS25; Sigma-Aldrich) and *2*) a *Staphylococcus aureus* lysate. The former was acquired directly from the supplier, while the latter was prepared by growing *S. aureus* overnight in Lysogeny broth (LB) growth media at 37°C, followed by centrifugation at 4000 RPM for 10 min and three consecutive washes with sterile ddH_2_O. The pellet was then resuspended in 500 µl of ddH_2_O, heat blocked at 80°C for 1 h and stored for 48 h in a −80°C freezer. The content was subsequently lyophilized and resuspended at a stock concentration of 1 mg/ml.

RAW264.7 murine macrophages were seeded in 96-well microplates at 10,000 cells/well and cultured for 24 h in unsupplemented RPMI medium. Cells were stimulated with 5 μg/ml of either *E. coli* LPS or the *S. aureus* lysate and simultaneously treated with each of the sugarcane-derived extracts diluted to a 1:8 ratio. Untreated but stimulated macrophages were used as positive control and untreated and unstimulated macrophages as negative control. After 48 h, all supernatants were collected and used immediately for quantifying the secreted cytokines.

#### Modeling Neuroinflammation

Neuroinflammation was modeled *in vitro* using the A53T mutant isoform of α-synuclein (S1071; Sigma-Aldrich), an endotoxin that pathologically aggregates in neurons and activates resident immune cells when secreted to the extracellular space (Harms et al., 2018). THP-1 human acute leukemia monocytes were stimulated with 10 μg/ml of A53T α-synuclein, to simulate monocyte infiltration during α-synucleinopathies, and simultaneously treated with each sugarcane-derived extract diluted to a 1:8 ratio. Untreated but stimulated monocytes were also used as positive control and untreated and unstimulated monocytes as negative control. After 48 h, all supernatants were collected, centrifuged to remove suspended cells, and used immediately for quantifying the secreted cytokines.

### Quantification of Secreted Cytokines and Chemokines

The Human Inflammation LEGENDPlex^™^ Panel (Biolegend, San Diego, CA, United States) was used to quantify the secretion of 13 cytokines and chemokines under each of the studied inflammation scenarios. Specifically, five pro-inflammatory cytokines (i.e., interleukin-1β (IL-1β), interferon-α2 (IFN-α2), tumor necrosis factor-α (TNF-α), monocyte chemoattractant protein (MCP-1), and interleukin-6 (IL-6)) and two anti-inflammatory cytokines (i.e., interleukin-33 (IL-33) and interleukin-10 (IL-10)) were analyzed given their pivotal roles in initiating, propagating and regulating inflammation (Zhang & An, 2007). The remaining five cytokines (i.e., interleukin-8 (IL-8), interleukin 17A (IL-17A), interleukin-12p70 (IL-12p70), interleukin 18 (IL-18), interferon-γ (IFN-γ)) are not analyzed since they were not detected in any of the evaluated scenarios. This bead-based assay follows the same basic principle of sandwich immunoassays and was performed following the manufacturer’s instructions. Briefly, antibody-conjugated beads were exposed to each supernatant for 2 h to promote binding of the specific cytokines/chemokines (if present). These were subsequently washed with 1X Wash Buffer and marked with biotinylated detection antibodies (DA) for one more hour. Finally, bead-analyte-DA sandwiches were fluorescently labeled with Streptavidin-phycoerythrin (SA-PE) for 30 min. Analyte-specific beads were separated by size and internal fluorescence intensity (APC) with the aid of a flow cytometer (BD FACSCanto II, BD Biosciences, NJ, United States). The separated bead populations with bound analytes were quantified according to their PE fluorescence. Acquired data was analyzed with the LEGENDPlex^™^ Data Analysis Software. Cytokine standard curves relating cytokine concentration to detected mean fluorescence intensity are found in Supplementary Figure S2.

### Gene Expression Analysis

The expression of seven genes involved in the TLR4 signaling pathways was evaluated before and after exposure to NCS extracts in the neuroinflammatory scenario. THP-1 monocytes were stimulated with A53T α-synuclein and simultaneously exposed to each of the NCS extracts for 4 h. Then, total RNA content was extracted from 5 × 10^5^ cultured cells using the Monarch MiniRNA Extraction Kit (New England Biolabs, MA, United States) according to the manufacturer’s instructions. Stimulated but untreated monocytes, as well as unstimulated monocytes, were also considered as positive and negative controls. Adequate RNA quality after extraction was determined spectrophotometrically by assuring that 260/280 and 260/230 ratios were above 2.0. Extracted RNA was reverse transcribed into cDNA using the OneScript Plus cDNA Synthesis Kit (Applied Biological Materials, Vancouver, Canada). Real-time qPCR was subsequently performed aided by the BrightGreen 2X qPCR MasterMix (Applied Biological Materials) and a Rotor-Gene Q Thermocycler (QIAGEN, Hilden, Germany). Primer pairs for genes involved in TLR4 signaling pathways were designed to have melting temperatures around 60°C and are listed in Supplementary Table S1. PCR runs included an initial 15 min period at 95°C for the heat inactivation of polymerases, followed by 40 amplification cycles. The relative fold gene expression (RFGE) was calculated according to the double delta Ct method described by Eqs 4, 5:

$$\text{RFGE}=2^{-{\text{C}}_{\text{t}}}$$ (4)

$$\Delta \Delta C_{t}={\left(C_{t}^{target\;gene}-C_{t}^{reference\;gene}\right)}_{sample}-{\left(C_{t}^{target\;gene}-C_{t}^{reference\;gene}\right)}_{control}$$ (5)

where the control sample was unstimulated monocytes and the reference gene was GAPDH, which is expressed constitutively.

### Statistical Analysis

All measurements are reported as mean ± standard deviation unless otherwise stated. Data analysis was performed with GraphPad Prism^®^ V 7.0a (GraphPad Software, La Jolla, CA, United States). Individual and grouped data sets were statistically compared through One-way or Two-way ANOVA, respectively. Multiple comparisons within treatments were performed with Tukey or Dunnet tests. Results with a *p*-value ≤ 0.05 were considered significant. *p*-values ≤ 0.05, 0.01, 0.001, 0.0001 were labeled as (*), (**), (***), (****), unless otherwise stated.

## Results and Discussion

**FIGURE 2:**
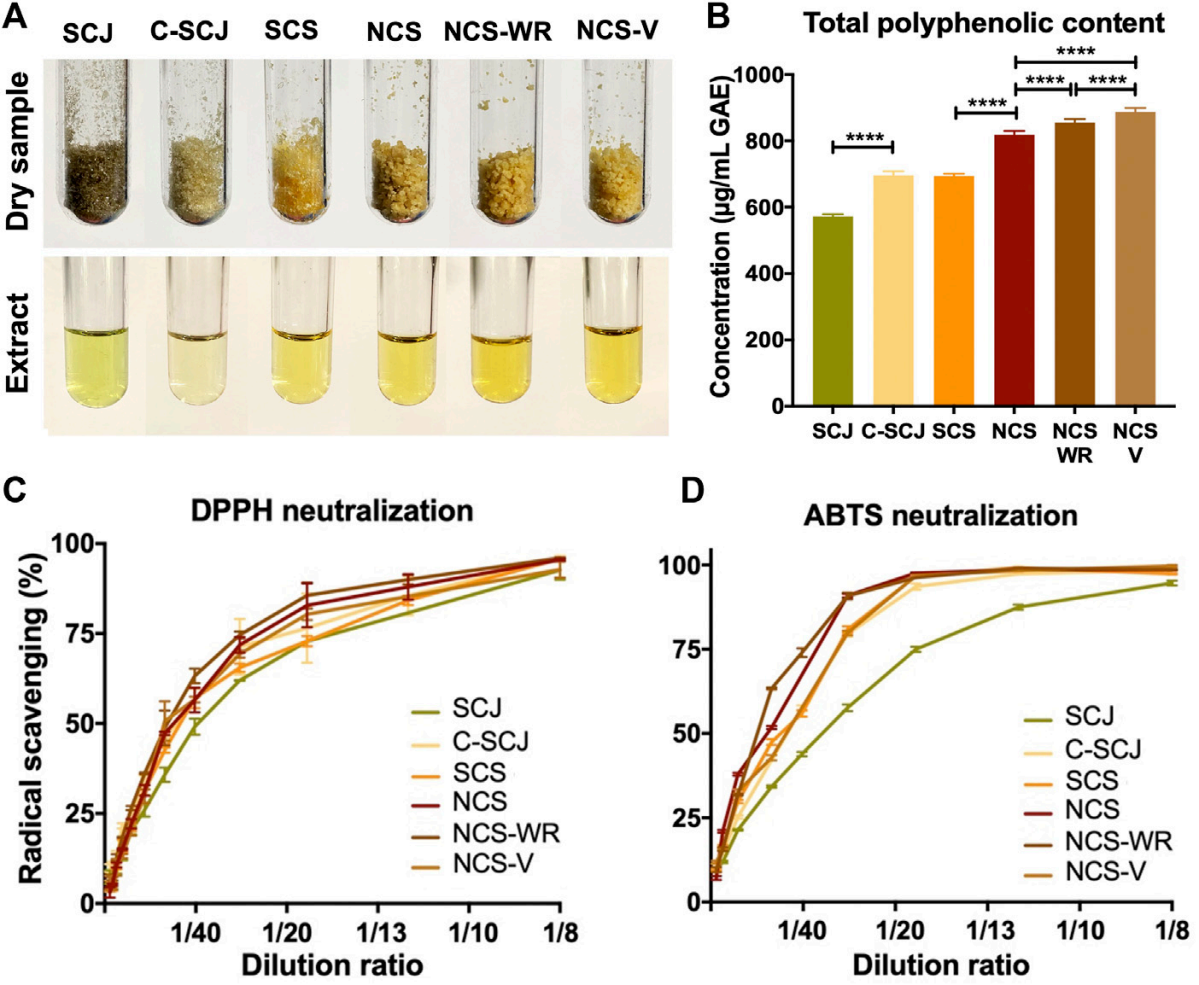
Polyphenolic profile of sugarcane-derived extracts. **(A)** Original dry samples and respective polyphenolic solutions after methanolic extraction. Extraction was conducted over the same dry weight for all samples and then concentrating the isolated fraction in a fixed volume of ddH_2_O. **(B)** Total polyphenolic content as determined by the Folin Ciocalteu method. Total polyphenol concentration is presented as an equivalent of gallic acid (GAE). (****) denotes statistical significance *p* < 0.0001. **(C)** Radical scavenging activity of sugarcane-derived extracts in the presence of DPPH and **(D)** ABTS radicals. Extract dilutions were prepared from stock solutions comprising the extracted fraction of a fixed sample dry weight reconstituted in a fixed volume. SCJ, Sugarcane juice; C-SCJ, clarified sugarcane juice; SCS, sugarcane syrup; NCS, non-centrifugal sugarcane; NCS-WR, NCS produced through window refractance evaporation; NCS-V, non-centrifugal sugarcane produced through vacuum-aided dehydration.

NCS is traditionally obtained from the thermal processing of sugarcane juice, the liquid phase extracted after milling sugarcane stems. This juice is subjected to three thermal processing stages: *1*) clarification, where temperature and vegetable flocculating agents are added to remove impurities, *2*) evaporation, where it is progressively dehydrated through constant heat exposure and *3*) concentration, where dehydrated syrup is crystalized by mechanical agitation. Here, we propose two modified evaporation methods that reduce exposure to extreme thermal processing conditions by *1*) reducing processing time (refractance window evaporation) and *2*) by reducing the process temperatures (vacuum-aided evaporation). Figure 1 shows a general schematic summarizing the traditional and modified NCS production processes. We collected samples from four different stages of the process to evaluate the impact of varying conditions on their biochemical profiles and bioactivity. Specifically, sugarcane juice (SCJ) obtained immediately after sugarcane milling, clarified SCJ (C-SCJ), sugarcane syrup (SCS), and the final NCS product, which was either traditionally produced or by employing any of the two proposed evaporation processes (NCS-WR and NCS-V). We then concentrated the polyphenolic compounds of each sample through methanolic solid-liquid extraction. To maintain composition-wise consistency with respect to the original samples, the process was conducted over the same dry weight for all samples and then concentrating the extracted content in an equal volume of water. Figure 2A shows the original samples and their corresponding extracts.

### Modified NCS Production Processes Increase Polyphenolic Yield

We determined the total polyphenolic content present in each sugarcane-derived extract through the Folin-Ciocalteu method (Lamuela-Raventós, 2017). It should be noted that the isolated polyphenols through methanolic extraction comprise mostly free polyphenolic compounds of the original sample given that polyphenols bound to other organic molecules exhibit decreased methanol solubility (Gulsunoglu et al., 2019). Accordingly, a proportional increase in free polyphenolic content was observed as sugarcane processing progressed along the stages (Figure 2B). This was most probably due to the occurrence of temperature-catalyzed bioconversion processes that favor the release of polyphenolic compounds from conjugated to free forms (Sharma et al., 2015). However, the polyphenolic content of the two NCS extracts produced under the two alternative concentration processes is significantly higher (*p* < 0.0001) than that of traditionally produced NCS. This suggests that, although initial thermal exposure is beneficial, excessive exposure may promote polyphenolic degradation and lead to suboptimal yields.

We corroborated these results by quantifying the most prominent polyphenols in each NCS extract with the aid of HPLC-coupled to mass spectrometry (Table 1). Of the 11 polyphenolic compounds identified, NCS-WR and NCS-V extracts exhibited a higher concentration in eight and in 11 of them with respect to traditional NCS. Moreover, each modified concentration method differentially contributed to preserving bioactive compounds, as evidenced by the marked concentration differences between specific compounds. For instance, theobromine and pelargonidin were best preserved during NCS-WR production, whereas for catechin and luteolin this was better achieved with NCS-V. Beyond these particular differences, all NCS extracts exhibited especially high concentrations of rosmarinic acid and cyanidin, two polyphenolic compounds with exceptional antioxidant and anti-inflammatory activities (Wang et al., 1999; Rocha et al., 2015). Given these marked polyphenolic differences, we proceeded to evaluate their effect over the antioxidant profiles and anti-inflammatory activities of the extracts, especially since the majority of the identified polyphenols have been individually correlated with immunomodulating properties (Aziz et al., 2018; Jeong et al., 2017; Kadioglu et al., 2015; Li et al., 2016; Martinez-Pinilla et al., 2015; Nakanishi et al., 2010; Nasri et al., 2017; Rocha et al., 2015; Soares et al., 2006; Wang et al., 1999; Wang and Cao, 2014).

**TABLE 1:** Identified polyphenolic compounds in each NCS extract through HPLC-MS.

Analyte	RT (min)	Concentration (mg/kg dry sample)					
		NCS	E (%)	NCS-WR	E (%)	NCS-V	E (%)
Theobromine	2.7	0.632	1	**1.894**	1	**0.812**	1
Catechin	3.1	1.264	1	**1.623**	1	**3.517**	1
Epicatechin	2.3	0.474	1	**0.541**	1	**0.812**	1
Quercetin	4.5	0.474	2	**0.812**	2	**0.812**	2
Rosmarinic acid	4.1	6.005	1	5.952	1	**8.658**	1
Luteolin	4.5	0.632	1	**1.082**	1	**1.894**	1
Kaempferol	5.0	0.632	0	0.271	0	**1.082**	0
Apigenin	4.9	0.316	3	**0.541**	3	**0.541**	3
Cyanidin	3.4	14.697	1	**17.856**	1	**16.504**	1
Kaempferol 3-glucoside	3.8	1.896	0	1.353	0	**2.706**	0
Pelargonidin	3.6	2.529	3	**6.493**	3	**2.976**	3
Total		29.552		**38.418**		**40.312**	

Bold numbers indicate that the concentration of that polyphenol is superior to that of traditional NCS. The respective chromatograms are shown in Supplementary Figure S3.

### All NCS Extracts Exhibit High, yet Comparable Chemical Radical Scavenging

The antioxidant activity of NCS products has been widely characterized, both *in vitro* and *in vivo*. Notably, NCS has shown antioxidant activities in human blood plasma as short as 60 min after consumption (Azlan et al., 2022) and stimulating effects over antioxidant enzymes (e.g., superoxide dismutase, catalase, glutathione peroxidase, and glutathione S-transferase) in mouse models (Kinjo et al., 2019; N. Singh et al., 2010). Accordingly, to evaluate if the modified production methods had any effect over the already described antioxidant potential of the resulting end products, we characterized the scavenging profiles of sugarcane-derived extracts in the presence of chemical radicals.

Figures 2C,D show that DPPH and ABTS radicals were effectively neutralized by all extracts in a concentration-dependent manner. The scavenging of these radicals increased nonlinearly with extract concentration, resembling a logarithmic behavior and saturating rapidly. Moreover, radical scavenging tended to be higher in extracts of NCS end-products with respect to less processed extracts. However, all NCS extracts presented similar radical scavenging profiles in the presence of both radicals, which contrasts with the previously observed differences in polyphenolic content between them. This suggests that the antioxidant profiles of NCS may be sufficiently robust to reflect compositional differences in polyphenolic compounds. In fact, all NCS extracts showed a comparable or superior scavenging of these radicals when compared to other plant-derived extracts (Floegel et al., 2011), as determined by their ascorbic acid equivalent radical scavenging per gram of dry sample (Table 2). Markedly, their ability to scavenge ABTS and DPPH radicals was superior to that of 48 and 40 of the most popular antioxidant-rich foods in the US, respectively (Floegel et al., 2011). Considering that the scavenging of ABTS radicals in the presence of all extracts was almost three times higher than the scavenging of DPPH radicals (Table 2), it is most likely that hydrophilic antioxidant compounds are predominantly contributing to the observed antioxidant profiles, given their superior affinity towards ABTS^+^ compared with DPPH^+^ (Kim et al., 2002; Floegel et al., 2011).

**TABLE 2:** Ascorbic acid equivalent (AAE) scavenging of DPPH and ABTS radicals by sugarcane-derived extracts.

Extract	Ascorbic acid equivalent radical scavenging (mg AAE/g dry sample)	
	DPPH	ABTS
SCJ	1.23 ± 0.04	2.03 ± 0.03
C-SCJ	1.24 ± 0.12	2.72 ± 0.04
SCS	1.26 ± 0.02	2.85 ± 0.02
NCS	1.43 ± 0.11	2.89 ± 0.01
NCS-V	1.39 ± 0.03	2.86 ± 0.01
NCS-WR	1.43 ± 0.06	2.84 ± 0.001

All extracts exhibited a significantly higher scavenging potential for the ABTS radical.

### Sugarcane-Derived Extracts Are Non-cytotoxic and Enhance Cellular Proliferation

**FIGURE 3:**
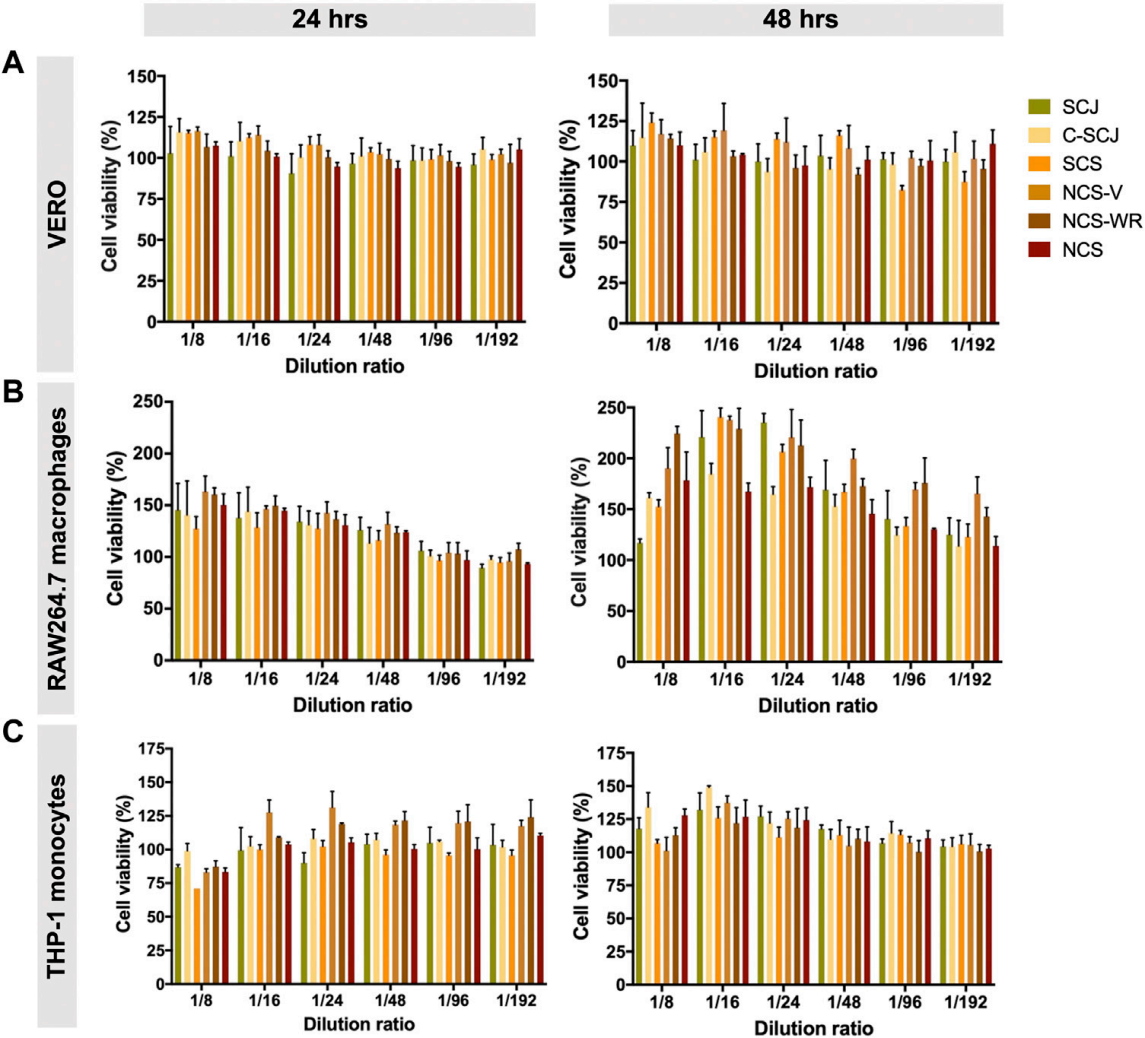
Cell viability of **(A)** Vero cells, **(B)** RAW264.7 macrophages and **(C)** THP-1 monocytes upon exposure to sugarcane-derived extracts for 24 and 48 h, as determined by the MTT assay. Extract dilutions were prepared from stock solutions comprising the extracted fraction of a fixed sample dry weight reconstituted in a fixed volume. All extracts maintain or even enhance cell viability with respect to untreated controls. SCJ, Sugarcane juice; C-SCJ, clarified sugarcane juice; SCS, sugarcane syrup; NCS, non-centrifugal sugarcane; NCS-WR, NCS produced through window refractance evaporation; NCS-V, non-centrifugal sugarcane produced through vacuum-aided dehydration.

We evaluated the cytocompatibility of all sugarcane-derived extracts by assessing their effect over the viability of Vero cells (10993-5 ISO Standard cell line for cytotoxicity assays) and two immune cell lines, namely THP-1 human acute leukemia monocytes and RAW264.7 murine macrophages, as determined by the MTT assay. We treated cells with six different extract concentrations that showed varied antioxidant profiles according to DPPH and ABTS assays, the highest of which exhibited 100% radical scavenging. Figure 3 shows that the viability of all treated cell lines was comparable or superior to untreated controls. Upon exposure to all extracts, Vero cells maintained metabolic activity levels above 100% irrespective of the extract concentration and exposure time (Figure 3A). Also, they exhibited ideal membrane integrity as determined by undetectable LDH leakage (Supplementary Figure S4). Accordingly, all extracts can be catalogued as non-cytotoxic according to ISO standards. Moreover, we observed that the viability of THP-1 monocytes and RAW264.7 macrophages increased with extract concentration (Figures 3B,C). This trend was especially distinguishable for the macrophages, whose viability increased by over 2-fold with respect to untreated controls upon exposure to the highest extract concentrations for 48 h. The superior viability observed for treated cells suggests that the bioactive composition of the extracts may be promoting proliferation in this type of immune cells. Overall, these results indicate that the developed extracts are cytocompatible and can be safely used further for evaluating the expected anti-inflammatory effect in both immune cell lines.

### Modified NCS Extracts Elicit Superior Anti-inflammatory Responses Than Traditional NCS

**FIGURE 4:**
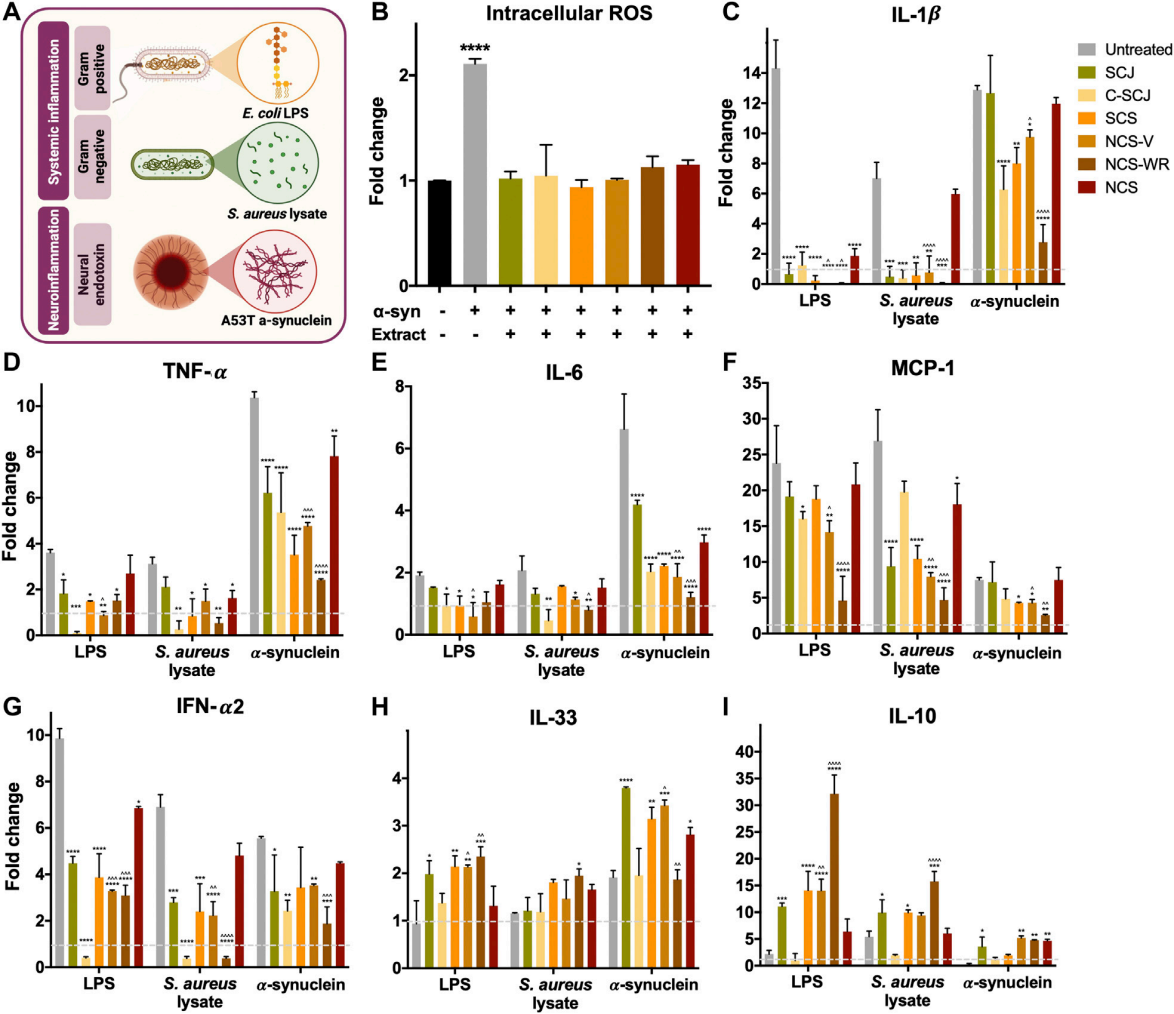
Anti-inflammatory effect of sugarcane-derived extracts. **(A)** Schematic illustration of the three stimulating agents employed for each considered inflammation scenario. Namely, LPS and an *S. aureus* lysate at 5 μg/ml dosages for modeling systemic inflammation through gram positive and negative bacteria in macrophages, and A53T α-synuclein at 10 μg/ml dosage for modeling neuroinflammation in infiltrating monocytes. Created with Biorender.com. **(B)** Intracellular ROS production in α-synuclein stimulated THP-1 monocytes in the presence and absence of sugarcane-derived extracts. All extracts significantly hamper ROS production in response to inflammatory stimuli. (*) denotes statistical significance with respect to untreated and unstimulated control. **(C)** Fold change secretion of IL- 1ß, **(D)** TGF-α, **(E)** IL-6, **(F)** MCP-1, **(G)** IFN-α2, **(H)** IL-33 and **(I)** IL-10 under each inflammation scenario in the absence and presence of sugarcane-derived extracts, with respected to unstimulated controls. Red labeled cytokines are pro-inflammatory and blue-labeled cytokines are anti-inflammatory. All extracts downregulate the secretion of pro-inflammatory cytokines and upregulate that of anti-inflammatory cytokines, especially modified NCS extracts. (*) denotes statistical significance with respect to stimulated but untreated controls, and (^) denotes significance with respect to traditional NCS.

Considering that all extracts demonstrated high radical scavenging profiles and cytocompatibility at the highest tested concentration, we proceeded to evaluate their anti-inflammatory potential at this concentration. We considered three inflammation scenarios to recapitulate inflammatory responses to different stimuli: *1*) systemic inflammation induced by gram positive or *2*) gram negative bacteria, and *3*) neuroinflammation induced by neural endotoxins (Figure 4A). Specifically, we stimulated RAW264.7 macrophages with *E. coli* LPS and an *S. aureus* lysate to model the first two scenarios, given the pivotal role of macrophages in initiating systemic inflammation (Fujiwara & Kobayashi, 2005). In contrast, THP-1 monocytes stimulated with the A53T mutant isoform of α-synuclein were used to model neuroinflammation, given that both *in vitro* and *in vivo* studies have demonstrated that infiltrating monocytes are essential for the initiation and propagation of neuroinflammatory environments in numerous neurological diseases (Baufeld et al., 2018; Harms et al., 2018; Spiteri et al., 2022). Moreover, A53T α-synuclein is known to form aggregates in solution, which have shown to unravel neurotoxic and inflammatory responses that characterize several neurodegenerative diseases (Stefanis, 2012). Previous reports have already shown that each of the selected stimulating agents are able to induce rapid activation of inflammatory pathways in the selected cell lines (Klegeris et al., 2008; Rutledge et al., 2012; Pidwill et al., 2021), which makes them suitable for modeling the selected inflammation scenarios *in vitro*.

Given that reactive oxygen species (ROS) are key mediators in the progression of inflammatory responses (Chelombitko, 2018), we first evaluated the ability of sugarcane-derived extracts for modulating intracellular ROS levels in stimulated immune cells (THP-1 monocytes). Considering that ROS production is a hallmark of inflammation, irrespective of the stimulus, we only evaluated ROS modulation in the neuroinflammation scenario. We stimulated and simultaneously exposed THP-1 monocytes to each extract and subsequently quantified intracellular ROS levels after 4 h. Figure 4B shows that all sugarcane-derived extracts significantly hamper the upregulation of ROS production in stimulated monocytes. Most importantly, ROS production under extract exposure was not significantly different to unstimulated controls, which indicates that they not only attenuate but halt the development of oxidative environments. Again, we failed to observe significant differences over the ROS modulating effects of NCS extracts, which is consistent with the results obtained for their radical scavenging activities.

To further characterize the anti-inflammatory and immunomodulatory effect of the sugarcane-derived extracts, we proceeded to quantify the secretion of five relevant pro-inflammatory cytokines (e.g., IL-1β, TNF-α, IL-6, MCP-1, IFN-α2) and two relevant anti-inflammatory cytokines (e.g., IL-33, IL-10) under each of the developed inflammation scenarios, both before and after treatment (Figures 4C–I). As expected, all stimulating agents significantly upregulated the production and secretion of pro-inflammatory cytokines, while that of anti-inflammatory cytokines remained comparable to unstimulated controls. Moreover, the extent of upregulation differed between stimulation scenarios, thus corroborating the anticipated ignition of differential inflammatory responses for each of them. However, secretion profiles changed significantly upon treatment with sugarcane-derived extracts, especially with the two derived from modified NCS (NCS-V and NCS-WR). For instance, the secretion of interleukin 1β (IL-1β), tumor necrosis factor-α (TNF-α) and IL-6 in systemic inflammation scenarios was reverted to values lower than or comparable to unstimulated controls upon treatment with NCS-V and NCS-WR extracts (Figures 4C–E). These two extracts also attenuated the secretion of these cytokines in the neuroinflammation scenario, although to levels still slightly higher than in unstimulated conditions. Most importantly, the secretion of IL-1β, TNF-α, and IL-6 under NCS-V and NCS-WR treatments was significantly lower than under traditional NCS treatments. These results are particularly promising given that these three cytokines play central roles in initiating inflammatory cascades by orchestrating the production of a wide array of other pro-inflammatory cytokines and chemokines (e.g., IL-8, monocyte chemotactic protein-1 (MCP-1), and IL-21) (Eskan et al., 2008; Parameswaran and Patial, 2010; Tanaka et al., 2014). Additionally, they oversee the recruitment of other immune cells (e.g., T-cells, B-cells, neutrophils) that are essential for directing host defense mechanisms (Zhang and An, 2007; Turner et al., 2014). We also evidenced the superior modulation of modified NCS extracts in the secretion of MCP-1 and interferon-α2 (IFN-α2) within all inflammation scenarios (Figures 4F,G). MCP-1 is a major cytokine participating in monocyte recruitment towards foci of active inflammation (Deshmane et al., 2009), whereas IFN-α2 is highly implicated in the activation and regulation of adaptive immune responses (Paul et al., 2015). Accordingly, the superior modulation of these pro-inflammatory cytokines by NCS extracts obtained through the modified concentration processes, suggests that their compositional differences with respect to NCS may be favoring superior anti-inflammatory responses.

The observed anti-inflammatory effect of sugarcane-derived extracts correlates well with their consistent upregulation of IL-33 and IL-10 secretion, two T helper 2 (Th2)-inducing cytokines closely related with anti-inflammatory responses (Figures 4H,I). These cytokines have been reported to attenuate immune responses by inducing the production of the anti-inflammatory cytokines IL-5 and IL-13 (Miller, 2011), inhibiting the release of pro-inflammatory mediators and decreasing antigen presentation (Iyer & Cheng, 2012). As observed earlier, the two NCS extracts obtained from modified processes also elicited a more prominent upregulation than NCS, which suggests that they are the most effective extracts at modulating inflammatory responses both in systemic and neuroinflammatory scenarios.

Despite the demonstrated superiority of both modified NCS extracts with respect to traditional NCS, NCS-WR exhibited a slightly superior modulating effect than NCS-V in most cases, especially over pro-inflammatory cytokine secretion in neuroinflammatory scenarios and anti-inflammatory cytokine secretion in systemic inflammation scenarios. In most cases, NCS-V exhibited a comparable effect to its immediate precursor (i.e., SCS), while the effect of NCS-WR was usually superior. This suggests that window refractance evaporation is not only preventing further polyphenolic degradation but may also be promoting temperature-catalyzed bioconversion processes that increase the level of free polyphenolic compounds. This is a reasonable assumption considering that it is not significantly reducing the employed temperature, as in vacuum-aided dehydration, but rather the exposure time to thermally degrading conditions.

However, considering the total polyphenolic content of NCS-V was slightly higher than NCS-WR, this suggests that there are specific polyphenols better preserved in NCS-WR that could be responsible for its anti-inflammatory superiority. In particular, theobromine, pelargonidin, and cyanidin, which were the most prominent and best-preserved polyphenols in NCS-WR, have been individually reported as potent anti-inflammatory agents (Jeong et al., 2017; Martinez-Pinilla et al., 2015; Wang et al., 1999). Most importantly, their reported anti-inflammatory effect was not solely associated to their antioxidant capacity but rather their interaction with inflammation signaling pathways. This is supported by the results shown in Figures 2C,D, 4B, which suggest that all NCS extracts elicit similar radical scavenging activities and intracellular ROS modulation despite their differential composition and effect over cytokine secretion. Therefore, we proceeded to evaluate the possible interaction of NCS extracts with the Toll-like receptor 4 (TLR4) signaling pathway, which plays crucial roles in initiating innate immune system responses towards bacterial infections and α-synucleinopathies (Lu et al., 2008; Fellner et al., 2013).

### Modified NCS Extracts Differentially Modulate TLR4 Signaling in Stimulated Monocytes

**FIGURE 5:**
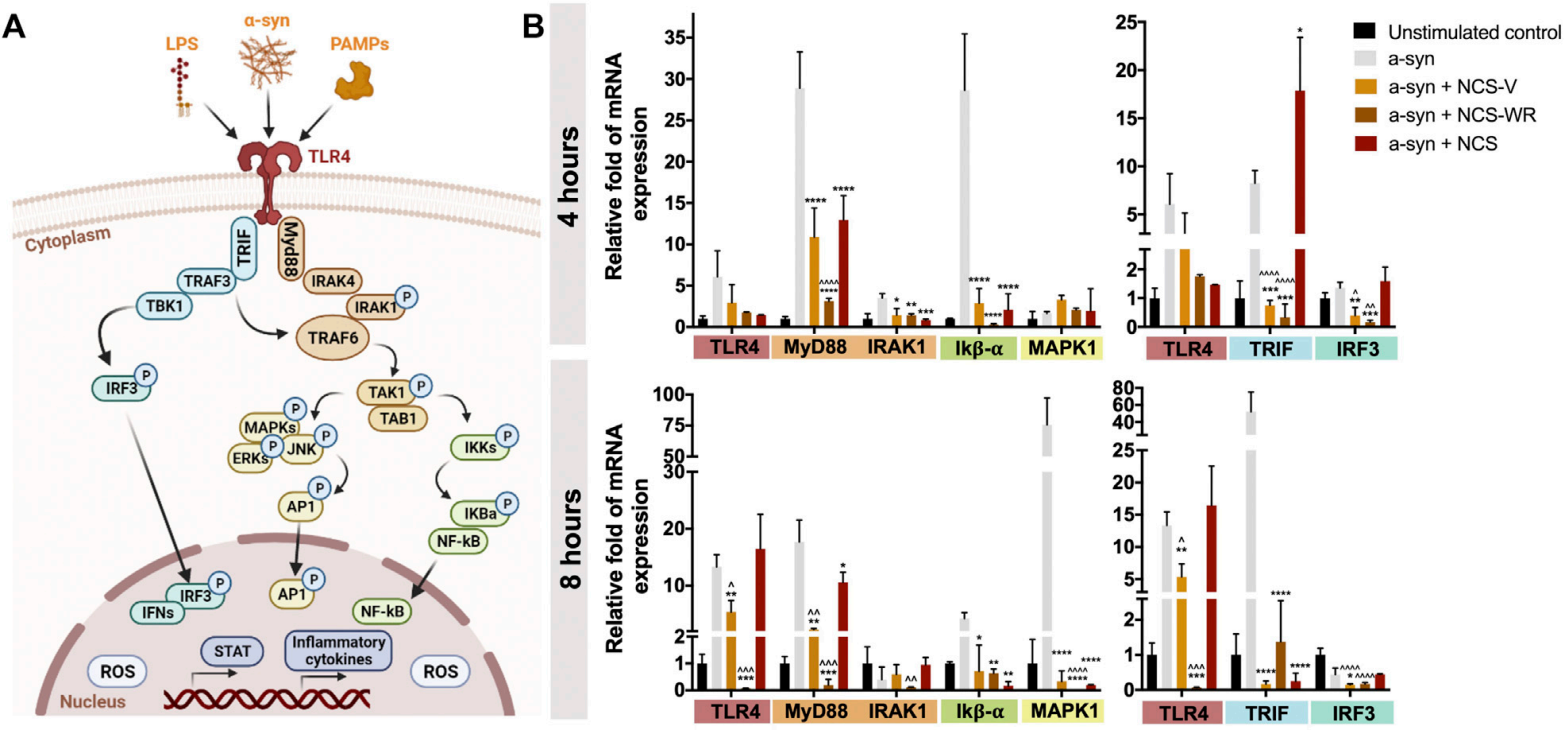
TLR4-signaling modulation by NCS extracts in α-synuclein stimulated monocytes. **(A)** Schematic of the three principal subpathways triggered by TLR4 activation. Ligand binding to TLR4 elicits two MyD88-dependent and one MyD88-independent responses, which result in the nuclear translocation of NF-kB, AP1 and IRF3. These three factors initiate inflammatory responses by promoting the transcription of inflammatory mediators and ROS production Created with BioRender.com. **(B)** Relative fold of mRNA expression of seven proteins involved in the three subpathways of TLR4 signaling (e.g., TLR4, MyD88, IRAK1, IkB- α , MAPK1, TRIF, IRF3) after 4 and 8 h of α -synuclein stimulation in the presence or absence of NCS extracts. All extracts attenuate the expression of these inflammatory markers, with NCS-WR eliciting a superior effect. (*) denotes statistical significance with respect to the stimulated but untreated control, and (^) denotes significance with respect to traditional NCS. TLR4, Toll-like receptor 4; Myd88, myeloid differentiation primary response protein 88; IRAK1/4, IL-1 receptor-associated kinase 1/4; TRAF3/6, TNF receptor associated factor 3/6; TAK1, Transforming growth factor beta-activated kinase 1; TAB1, TAK1 Binding Protein 1; IKK, Inhibitor of Kappa B Kinase; IKB α , inhibitor of kappa B- α ; NF-kB, nuclear factor kappa B; MAPK, mitogen-activated protein kinase; JNK, c-Jun N-terminal kinases; ERK, extracellular signal-regulated kinase; AP1, activator protein 1; TRIF, TIR-domain-containing adaptor-inducing interferon- β ; TBK1, TRAF Family Member Associated NF-kB Activator-binding kinase 1; IRF3, interferon regulatory factor 3; IFN, interferon; STAT, Signal Transducer and Activator of Transcription.

Polyphenols have been traditionally studied as potent regulators of inflammation due to their reported interference with TLR4 signaling (Rahimifard et al., 2017), a key pathway in propagating innate immune responses in chronic inflammatory disorders by orchestrating the production or activation of pro-inflammatory mediators (e.g., pro-inflammatory cytokines and chemokines, ROS, inducible nitric oxide synthase (iNOS) and inducible cyclooxygenase-2 (COX-2)) (Lucas and Maes, 2013; Kuzmich et al., 2017)Lucas and Maes, 2013. Figure 5A shows a general schematic of the intracellular subpathways triggered by TLR4 activation, which can result from its interaction with exogenous molecules (e.g., LPS, pathogen-associated molecular patterns (PAMPs)) or endogenous molecules secreted by damaged cells (e.g., α -synuclein) (Akira et al., 2006; Koedel et al., 2007; Codolo et al., 2013). Briefly, three subpathways can be identified upon TLR4 activation: myeloid differentiation primary response protein 88 (MyD88) dependent responses, which promote the nuclear translocation of *1*) the nuclear factor kappa B (NF-kB) and *2*) the activator protein 1 (AP-1), and MyD88 independent responses, which promote the nuclear translocation of *3*) the interferon regulatory factor 3 (IRF3) (Rahimifard et al., 2017). Upon activation, these three intracellular routes orchestrate the activation of several pro-inflammatory mediators that, among others, promote inflammatory cytokine transcription and ROS production.

Accordingly, we evaluated the change in mRNA levels of seven checkpoint proteins involved in these three intracellular routes during the first 8 h of exposure to TLR4 ligands and in the presence or absence of each NCS extract. Specifically, MyD88, IL-1 receptor-associated kinase 1 (IRAK1), inhibitor of kappa B- α (IkB- α ) and mitogen-activated protein kinase 1 (MAPK1) were selected to evaluate the two subpathways of MyD88-dependent responses. Conversely, the TIR-domain-containing adaptor-inducing interferon- β (TRIF) and the IRF3 were selected to evaluate MyD88-independent responses. Finally, the overall impact on this signaling cascade was assessed by quantifying TLR4 expression. The transcription of these checkpoint proteins is expected to increase during the initial response to inflammatory stimuli, given that these signaling cascades employ feedforward mechanisms to increase the availability of relevant proteins and enhance the corresponding intracellular responses. Accordingly, we assessed the modulating effect of each NCS extract over the elicited inflammatory responses by evaluating differential changes in mRNA levels with respect to stimulated and unstimulated controls in the absence of extracts. Although we only perform this analysis on our neuroinflammatory scenario (i.e., α-synuclein stimulated monocytes), it exemplifies possible interactions with this signaling route irrespective of the activating stimulus, given that analogous intracellular responses can be elicited from diverse TLR4 ligands.

As expected, α -synuclein stimulation effectively induced the overexpression of all TLR4-induced signaling routes in a time-dependent manner (Figure 5B). The TLR4-MyD88-NFkB pathway was significantly upregulated after only 4 h of exposure, as evidenced by the almost 30-fold increase in MyD88 and IkB- α expression. However, the upregulation of TLR4-MyD88-AP1 and TLR4-TRIF-IRF3 pathways was only evident after 8 h of exposure, with MAPK1 and TRIF reaching over 70-fold increases in expression. Consistent with previous results, the exposure to all NCS extracts significantly attenuated the observed upregulation in TLR4 signaling, with NCS-WR eliciting the most significant effects. For instance, the previously observed upregulation in TLR4-MyD88-IkB- α signaling after 4 h was considerably halted by NCS-WR, as evidenced by the six-fold reduction in MyD88 expression, the maintenance of IRAK1 at basal expression levels and the drastic downregulation of IkB- α with respect to the unstimulated control. Although NCS-V and traditional NCS also attenuated this signaling route, their effect was not as marked as that for NCS-WR. A similar pattern can be observed for TLR4-MyD88-AP1 signaling, whose expected upregulation after 8 h was also halted by NCS-WR exposure. In particular, MAPK1 expression was significantly downregulated to barely detectable levels, and was considerably lower than that elicited by the other NCS extracts. This could suggest that polyphenols that were best preserved in NCS-WR may be contributing to the observed differences. Theobromine, for example, has been previously reported to block NF-kB activation by forming inhibitory complexes with IkB- α /NF-kB (Rahayu et al., 2016), which correlates well with the fact that IkB- α expression upon exposure to NCS-WR fell considerably below basal levels. Similarly, cyanidin has been reported to inhibit the phosphorylation of extracellular signal-regulated kinase 1 (ERK1), MAPK1 and c-Jun N-terminal kinases (JNK) (Nam et al., 2016), which significantly compromises the TLR4-MyD88-AP1 pathway. Other prominent polyphenols in NCS-WR such as pelargonidin have also shown to intervene with MyD88-dependent signaling (Hämäläinen et al., 2007; Lee et al., 2018), although their inhibitory mechanisms remain unclear. However, further studies are needed to decipher the specific contribution of each compound to the observed anti-inflammatory effects, as well as to elucidate possible synergistic relationships between compounds. Regardless, the superior modulation of MyD88-dependent signaling routes by NCS-WR is consistent with the previously observed results in cytokine secretion modulation, especially since these routes have been reported to be the most important for initiating inflammatory responses (Rahimifard et al., 2017).

Despite the observed differences in modulating MyD88-dependent signaling, NCS-WR and NCS-V elicited similar effects over TLR4-TIRF-IRF3 signaling. In particular, the observed overexpression of TRIF, which peaked at 8 h after stimulation, was maintained below basal levels in the presence of both extracts. Notably, traditional NCS showed no interference with the initial increase in TRIF expression during the first 4 h, but eventually reduced its expression to levels comparable to the NCS extracts obtained by the modified processes. We observed a similar pattern for IRF3 expression, however, further phosphorylation analyses should be considered as future work to corroborate this finding, given that its expression in untreated controls did not vary as significantly as for the other protein markers. This could be related to the fact that IRF3 is constitutively expressed and is dynamically activated during the TLR4 signaling cascade (Au et al., 1998). Finally, it is important to note that TLR4 expression under traditional NCS treatment remained at the same level of stimulated but untreated controls, while such expression was reduced significantly under modified NCS treatments. This corroborates that the overall effect of NCS extracts obtained by the modified processes was superior to traditional NCS, positioning these extracts as superior therapeutic products with strong potential for the modulation of chronic inflammatory disorders.

## Conclusion

The growing interest in the anti-inflammatory effects of plant-derived products for tackling chronic inflammation disorders has encouraged the development of novel production methods that favor phytochemical compositions and bioactivities for therapeutic purposes. Our results demonstrate that reducing exposure to extreme thermal processing conditions during NCS production, both by decreasing the employed temperatures and exposure times, effectively improves the retention of polyphenolic compounds. Although these compositional differences showed no impact on the already proven high antioxidant capacity and cytocompatibility of NCS, they do translate into a superior anti-inflammatory potential as demonstrated by marked reductions in pro-inflammatory cytokine secretions and enhanced anti-inflammatory cytokine secretions, when tested in both systemic and neuroinflammatory scenarios *in vitro*. Most importantly, we showed that these differences are directly reflected in the ability of modified NCS to interfere with TLR4 signalling in human monocytes, although phosphorylation analyses of implicated proteins are needed to detect specific targets within this inflammation pathway. Even though both types of modified NCS extracts elicit superior anti-inflammatory effects than traditional NCS, our results consistently demonstrated that reducing exposure time to extreme processing conditions is more effective in terms of bioactivity results. However, this should be corroborated further *via in vivo* inflammation studies to fully characterize the potential of the proposed NCS extracts. Moreover, we want to emphasize that, despite these promising results, the used NCS extracts purposefully concentrate the bioactive molecules of interest and, therefore, their observed anti-inflammatory effects may overestimate those of whole NCS products. Future work should be therefore focused on the rational development of edible NCS-derived products that concentrate the optimized polyphenolic profiles obtain here to ultimately enhance nutraceutical and pharmacological benefits directly related to preventing and therapeutically resolving chronic inflammation.

## Data Availability

The original contributions presented in the study are included in the article/Supplementary Material, further inquiries can be directed to the corresponding authors.
