# Supplementary material for: Evaluating the Impact of Thermal Processing on the Anti-Inflammatory Activity of Non-Centrifugal Cane Sugar: Implications on Cytokine Secretion and TLR4 Signaling
Source: Front Pharmacol. 2022 Jun 28;13:905347. doi: 10.3389/fphar.2022.905347 (PMC9274305; doi:10.3389/fphar.2022.905347)
Supplement: Supplementary file 1 [file DataSheet1.PDF]

Supplementary Material

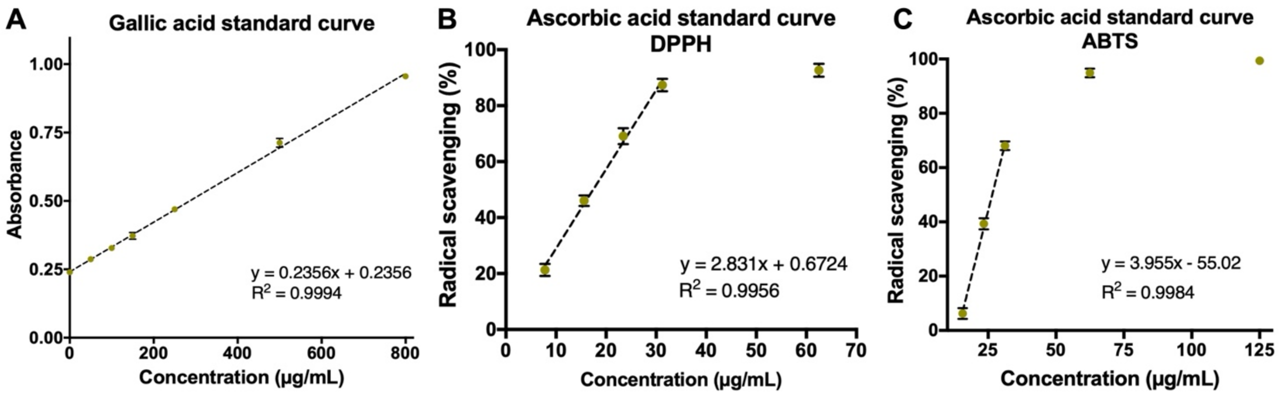

Supplementary Figure 1. (a) Standard curve correlating absorbance with gallic acid concentration. Used to estimate the total polyphenolic content in each extract as gallic acid equivalent concentration. (b) Standard curve correlating DPPH and (c) ABTS radical scavenging percentage with ascorbic acid concentration. Considering radical scavenging saturates after certain concentration, interpolation was performed on the linear region of each curve and the ascorbic acid equivalent antioxidant capacity of each extract was calculated at extract concentrations whose radical scavenging fell within these regions.

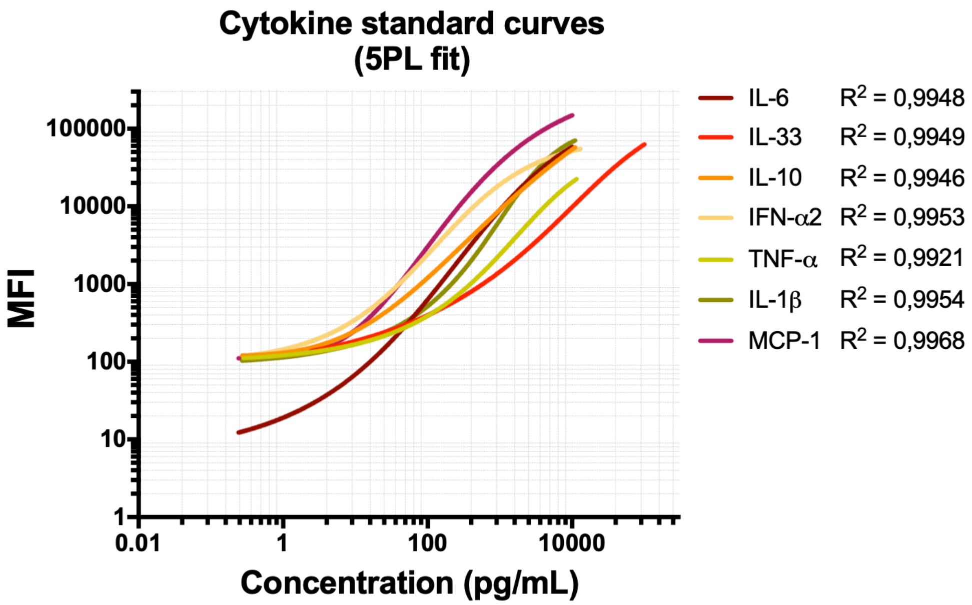

Supplementary Figure 2. Standard curves correlating mean fluorescence intensity (MFI) with cytokine concentration, as determined by the LEGENDPlex Human Inflammation Panel. Standard curves were calculated by fitting a five-parameter logistic curve (5PL) for the pro-inflammatory cytokines IL-6, IFN-a2, TNF-a, IL-1b, MCP-1 and anti-inflammatory cytokines IL-33 and IL-10.

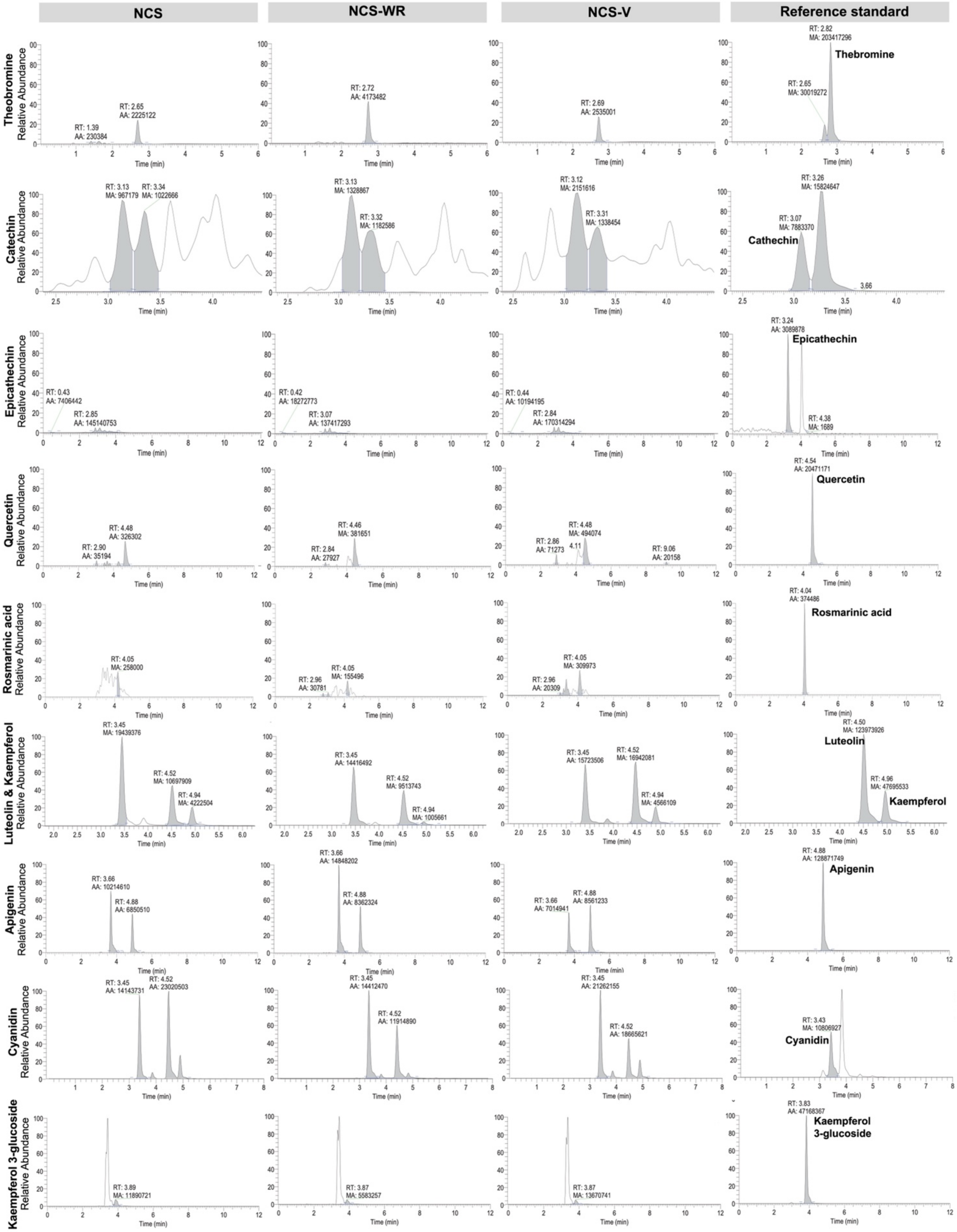

NCS
NCS-WR
NCS-V
Reference standard
Theobromine
Catechin
Epicatechin
Quercetin
Rosmarinic acid
Luteolin & Kaempferol
Apigenin
Cyanidin
Kaempferol 3-glucoside
Relative Abundance
Time (min)
Thebromine
Cathechin
Epicathechin
Quercetin
Rosmarinic acid
Luteolin
Kaempferol
Apigenin
Cyanidin
Kaempferol 3-glucoside

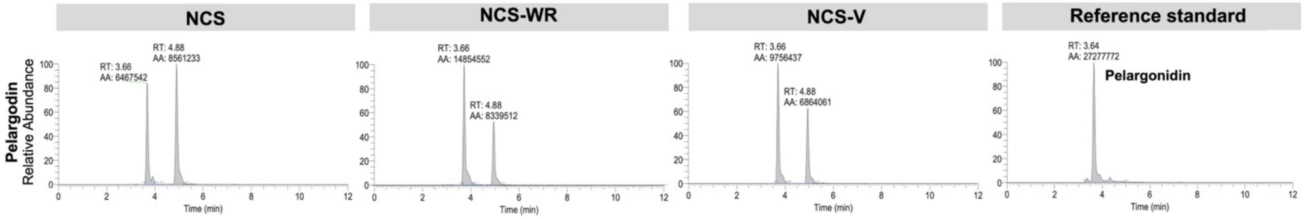

Supplementary Figure 3. Chromatograms of eleven polyphenolic compounds that predominate in NCS, NCS-WR and NCS-V samples obtained from HPLC coupled to mass spectrometry. Reference standards for each polyphenol were used for polyphenolic identification.

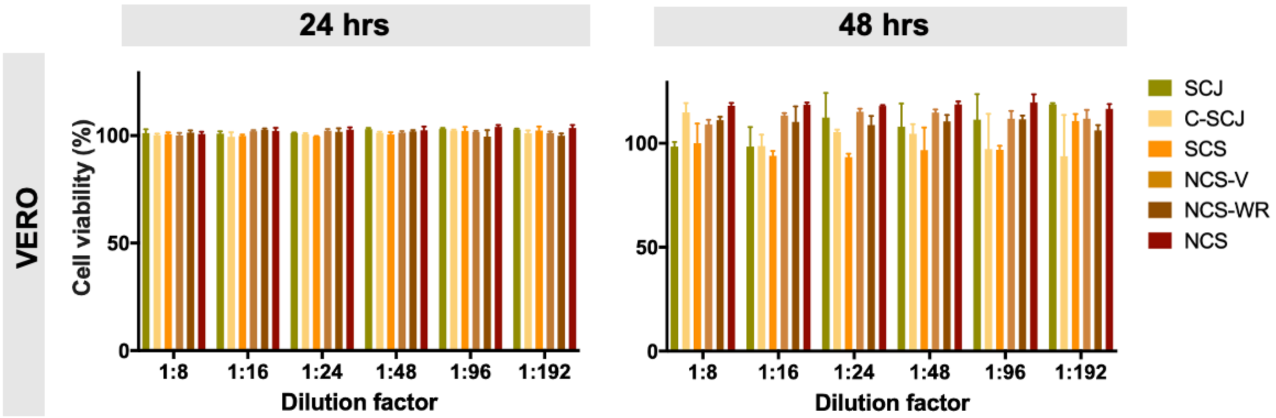

Supplementary Figure 4. Cell viability of VERO cells exposed to sugarcane-derived extracts for 24 and 48 h, as determined by LDH leakage. Viability values at or above 100% indicate that LDH content in cultured supernatants was comparable or inferior to untreated controls.

Supplementary Table 1. Primer sequences designed and implemented for assessing TLR4 signaling in THP-1 human monocytes.

Target	Forward sequence	Reverse sequence
TLR4	CACCTGATGCTTCTTGCTG	TCCTGGCTTGAGTAGATAA
MyD88	GCTTGGGCTGCTTTTCATT	CCTGCTCACATCATTACAGT
TRIF	GCACCAACTACCCAGTGGA	TGGCGTCTGGTCTTTGACAG
IRAK1	TGAAGAGGCTGAAGGAGAA	CACAATGTTTGGGTGACGAA
IRF3	TCTGCCCTCAACCGCAAAGAAG	TACTGCCTCCACCATTGGTGTC
IKB-α	GCTGAAGAAGGAGCGGCTACT	TCGTACTCCTCGTCTTTCATGGA
MAPK1	TCAAGCCTTCCAACCTG	GCAGCCTACAGACCAAA
